# De Novo Renal Cell Carcinoma in Kidney Transplant Recipients: Incidence, Outcomes, and Therapeutic Challenges

**DOI:** 10.3390/cancers17132200

**Published:** 2025-06-30

**Authors:** Jacob Schmidt, Malte Lehnert, Isabel Lichy, Henning Plage, Jonathan Jeutner, Lukas Kurz, Bernhard Ralla, Markus H. Lerchbaumer, Thorsten Schlomm, Frank Friedersdorff, Andreas Maxeiner, Robert Peters

**Affiliations:** 1 Charité—Universitaetsmedizin Berlin, Corporate Member of Freie Universitaet Berlin, Humboldt-Universitaet zu Berlin, and Berlin Institute of Health, Department of Urology, 12203 Berlin, Germany; 2 Charité—Universitaetsmedizin Berlin, Corporate Member of Freie Universitaet Berlin, Humboldt-Universitaet zu Berlin, and Berlin Institute of Health, Department of Radiology, 10117 Berlin, Germany; 3 Department of Urology, Evangelisches Krankenhaus Koenigin Elisabeth Herzberge, 10365 Berlin, Germany

**Keywords:** kidney transplantation, renal cell carcinoma, immunosuppressive therapy, oncological outcomes, propensity score matching

## Abstract

**Background/Objectives**: Kidney transplantation is associated with an increased risk of renal cell carcinoma (RCC). This study aimed to evaluate the outcomes of de novo RCC in kidney transplant recipients (KTRs). **Methods**: We retrospectively identified 50 de novo RCC cases among 4012 KTRs transplanted from 2005 to 2024. Data on patient characteristics and outcomes were collected. Propensity score matching (PSM) compared 34 localized RCC cases in KTRs with 34 non-transplant RCC cases. The statistical analyses used Kaplan–Meier estimates, the log-rank test, and the Cox regression. **Results**: The RCC incidence was 0.64 per 1000 person-years, with a standardized incidence ratio of 4.40 (95% CI: 3.33–5.80). In the KTR cohort, clear cell RCC was present in 42%, and papillary RCC was present in 42%. RCC developed predominantly in native kidneys (92%). UICC stage I was present in 74%. The treatment for the non-metastatic RCC was nephrectomy in the majority of cases (91%). For the metastatic RCC, 71% received a tyrosine kinase inhibitor (TKI). In the KTR cohort, the 3- and 5-year overall survival (OS) rates were 85% and 72%, respectively, with a median OS of 199 months; the synchronous metastasized (M1) patients had a median OS of 14 months. Rejection, age, advanced UICC stage, higher pT stage, clinical positive lymph nodes, M1, and higher grade were significantly associated with poor OS. The 5-year OS (96% vs. 84%, *p* = 0.72) and MFS (92% vs. 93%, *p* = 0.61) were comparable in the PSM cohort between the KTRs and the non-KTRs in the localized RCC. **Conclusions**: KTRs have a higher risk of RCC and present at a localized stage with comparable OS rates to non-transplant RCC patients. Adverse tumor characteristics, including synchronous metastases, significantly affect the prognosis, highlighting the need for surveillance and individualized treatment, particularly for metastatic RCC.

## Simple Summary

Kidney transplant recipients have a higher risk of developing kidney cancer than the general population, particularly in their native kidneys. This study examines the prevalence of kidney cancer in transplant patients, as well as the clinical presentation, risk factors, available treatment options, and long-term outcomes for patients and grafts. Our research showed that most cases are diagnosed at an early stage, so that effective treatment is possible, and survival rates are comparable to those of non-transplant patients. However, the prognosis becomes less favorable when the cancer is advanced or has metastasized. These results underline the importance of continuous monitoring of the transplant and the native kidney and the need for personalized treatment strategies to optimize the outcomes for transplant patients with kidney cancer.

## 1. Introduction

Kidney transplantation (KT) is considered the therapeutic gold standard for end-stage renal disease (ESRD), and it significantly improves the quality of life for recipients compared with dialysis [1]. Kidney transplant recipients (KTRs) have been reported to exhibit a 3- to 5-fold increased risk of cancer compared to the general population, with genitourinary cancers accounting for the majority of all non-cutaneous cancers [2,3]. Renal cell carcinoma (RCC) is the most common genitourinary malignancy in KTRs, with a reported incidence of 0.58–0.93%, representing a 5- to 10-fold increase compared to the general population [4]. The most common histological subtypes are clear cell renal cell carcinoma (ccRCC), followed by papillary renal cell carcinoma (pRCC) [5]. RCC in KTRs may develop in either the native kidneys or the graft, potentially aggravating the oncologic therapy and clinical management [6]. The therapeutic options vary based on the tumor stage, the location, and the delicate balance required to maintain graft function [7]. For a RCC that occurs in the native kidney, radical nephrectomy remains the standard treatment. In cases of graft RCC, a nephron-sparing surgery is generally preferred, though its feasibility depends on the tumor’s size and characteristics [8]. For metastatic RCC, no definitive consensus has been established; the current treatment options include targeted therapies, such as tyrosine kinase inhibitors (TKIs), and immunotherapy [7]. Overall, the clinical data on RCC outcomes in KTRs remain sparse, and many questions regarding the complexities inherent in managing RCC in KTRs—ranging from surgical decision-making to the integration of systemic therapies—remain to be answered. While the increased incidence and typical presentation of RCC in KTRs have been described, the long-term survival data, transplant-specific prognostic factors, and direct comparisons with non-transplant RCC patients are limited. Our study addresses these gaps by providing extended follow-up data, analyzing transplant-related risks, and comparing outcomes with non-KTR patients.

The present single-center retrospective study aims to provide a comprehensive analysis of de novo RCC in KTRs, offering insights into clinical features, therapeutic strategies, risk factors, and prognosis. In addition, we performed a propensity score-matched comparison with a non-KTR RCC cohort to evaluate whether the presence of a renal graft leads to poorer survival in patients with localized tumors.

## 2. Materials and Methods

### 2.1. Patients

A total of 4012 patients who had undergone a KT at the Charité Hospital in Berlin (Campus Charité Mitte and Campus Virchow-Klinikum) between January 2005 and May 2024 were assessed for the occurrence of RCC after the KT, and 50 cases were identified. Patients with pre-existing RCC before the KT or with multi-organ transplants (e.g., kidney–pancreas) were excluded from the analysis. However, patients undergoing second or subsequent kidney transplantations were included. If a histopathological confirmation was not available, patients were included based on characteristic radiological findings suggestive of RCC in combination with a clinical assessment and multidisciplinary tumor board consensus. This applied only when no prior history of RCC existed, and the tumor was located in the native kidney. All donor kidneys had undergone a standard assessment: deceased donor organs were inspected macroscopically during their procurement, and all living donors received preoperative contrast-enhanced CT imaging. At our center, KTRs are followed in a standardized long-term care program post KT, which includes clinical visits, laboratory testing, and abdominal ultrasonography, typically performed annually. No structured RCC screening with a CT or MRI is routinely implemented in asymptomatic patients. Demographic information (gender, age, BMI, donor gender and age), information on the patient’s medical history (primary kidney disease, pre-existing conditions, maintenance immunosuppression regime, waiting time for KT, time on dialysis), surgical details (cold ischemia time (CIT), operative time), oncologic features and pathological reports (tumor stage, grading, histological subtype, clinical lymph node status, metastases), therapies, and outcomes (graft survival; GS: time between KT and graft failure, overall survival; OS: time between tumor diagnosis and death, recurrence-free survival; RFS: time between tumor diagnosis and recurrence, metastasis-free survival; MFS: time between tumor diagnosis and metachronous metastases) were collected from the medical records. Complications occurring after the KT were classified according to the Clavien–Dindo classification (CDC) in the first 30 postoperative days [9]. The clinical lymph node status and the presence of metastases were assessed in staging imaging using computed tomography or magnetic resonance imaging. For the comparison with non-KT RCC patients, propensity score matching (PSM) was performed between the KTRs with localized RCC and a cohort of 845 non-transplanted RCC patients who had undergone nephrectomy or partial nephrectomy at the Charité in Berlin between January 2008 and November 2014. The available clinical data and histopathological data of the non-transplanted RCC cohort included age, histological subtype, grade, and tumor stage (pT), as well as clinical follow-up data.

### 2.2. Statistical Analysis

The statistical analysis was performed using IBM SPSS Statistics 29 (Armonk, NY, USA), Posit Software RStudio 2025.05 (Boston, MA, USA), and SAS Institute Inc. JMP Pro 18 (Cary, NC, USA). The propensity score matching (PSM) was used to adjust for differences between the non-KT RCC cohort and the KT-RCC cohort, excluding patients with synchronous metastases. After excluding 305 cases due to missing values, 583 cases remained. A 1:1 PSM with the caliper set at 0.01 was performed, incorporating age, pT stage, grade, and histological subtype (ccRCC, pRCC, and others), which resulted in 68 matched cases. The standard mean difference as well as Mann–Whitney U tests were used for the analysis of the continuously coded variables, and a Chi-square test for multiple nominal variables was used to compare the patient characteristics and oncologic outcomes between KTRs and non-KTRs with RCC. The standardized incidence ratio (SIR) was calculated by dividing the observed RCC cases by the expected cases, based on the European population-based incidence rates for renal cell carcinoma according to Möller et al. [10]. The OS, MFS, and GS were determined using the Kaplan–Meier method and log-rank testing. The restricted mean survival time (RMST) analyses for the OS and MFS were conducted using truncation times of 60 and 120 months. Univariate Cox regressions were used to analyze the relationship between OS and patient characteristics (age, gender, BMI, immunosuppression, tumor history, smoking history, waiting time, delayed graft function, history of rejection, graft failure, living donor, HLA mismatches, percentage of reactive antibodies (RPAs)), and tumor characteristics (histological subtype, T-stage, UICC stage, nodal and metastatic status, rejection history, tumor grade, tumor location, recurrence). We defined *p*  <  0.05 to indicate statistical significance.

## 3. Results

### 3.1. Kidney Transplant-Specific Patient Characteristics

As shown in Table 1, the median age at KT was 56.5 years (IQR: 45.25–63.25), with a median BMI of 23.9 kg/m^2^ (IQR: 22.61–27.29). Thirty-five (70%) patients were male. Twelve (24%) patients had undergone a living donor KT. The most common underlying CKD causes are shown in Table 1.

The induction immunosuppressive regimen included Basiliximab (84%), Mycophenolate mofetil (MMF) (90%), and others (6%). The maintenance immunosuppressive regimen included MMF (94%), tacrolimus (64%), corticosteroids (52%), and cyclosporine (20%). The median waiting time for a KT was 36.5 months (IQR: 10.75–77.25), and the median duration of dialysis was 62.5 months (IQR: 21.5–88.75). Forty-six (92%) patients had hemodialysis, and 3 (6%) had peritoneal dialysis. The median follow-up after the KT was 139 months (IQR: 88.25–175).

### 3.2. Graft Function and Postoperative Complications After Kidney Transplantation

Five (10%) patients experienced CDC ≥ 3 complications, 20 (40%) had delayed graft function (DGF), and 15 (30%) experienced a graft rejection during follow-up (Table 1). The median creatinine levels decreased from 7.1 mg/dL (IQR: 5.86–9.24) preoperatively to 1.5 mg/dL (IQR: 1.22–2.03) at 6 months post KT and remained stable at 1.6 mg/dL at 5 years (IQR: 1.20–2.04). Twenty-two (44%) patients experienced a graft failure (GF), with a median GS of 190 months (95% CI: 164–216). As shown in Table 1, the main reasons were death with functioning graft (13%), chronic rejection (10.9%), and tumor-associated causes (4.3%).

### 3.3. Oncologic Features and Tumor-Specific Patient Characteristics

Among 4012 patients, 50 (1.25%) developed RCC during the follow-up. Therefore, the incidence in our cohort was 0.64 cases per 1000 person-years, and the SIR was 4.40 (95% CI: 3.33–5.80). The histopathological analysis identified ccRCC in 21 (42%), pRCC in 21 (42%), and mixed ccRCC and pRCC (4%). The SIR for ccRCC was 2.31 (95% CI: 1.51–3.54), while for pRCC, it was 12.32 (95% CI: 8.03–18.89). Other histological subtypes, including chromophobe, sarcomatoid, and tubule-cystic RCC, were found in three (6%) cases (Table 2). RCC was detected in the native kidney in 46 (92%) patients and in the graft in 4 (8%) cases. The median age at the RCC diagnosis was 58.5 years (IQR: 51–68.25), with a median time from the KT to RCC of 47 months (IQR: 13.25–83.5). Among the four cases of RCC arising in the graft, the median time from the KT to the tumor diagnosis was 126 months (range: 95–143 months). A prior history of malignancy was present in 11 (22%) patients, and 18 (36%) had a history of smoking.

The pathologic staging classified pT1a (56%), pT1b (18%), and pT3a (10%), but no pT2 tumors (Table 2). Positive lymph nodes in the staging imaging were documented in eight (16%) patients. Seven (14%) patients had synchronous metastases, primarily in the lungs (85.7%), lymph nodes (71.4%), and bones (57.1%). UICC stage I was present in 37 (74%) patients, III in 6 (12%) patients, and IV in 7 (14%) cases.

### 3.4. Oncologic Outcomes

The treatment for non-metastatic RCC included native nephrectomy in 36 (83.7%) cases or transplant nephrectomy in three (7%) cases with graft RCC, local ablation in one case, native nephrectomy and systemic therapy with a TKI, and no treatment due to the patient’s decision in one case, respectively (Table 3). For metastatic RCC, the TKI was administered in four patients (57.1%), native nephrectomy was performed in two cases (28.6%), and a combination of both was performed in one patient (14.3%).

**Figure 1:**
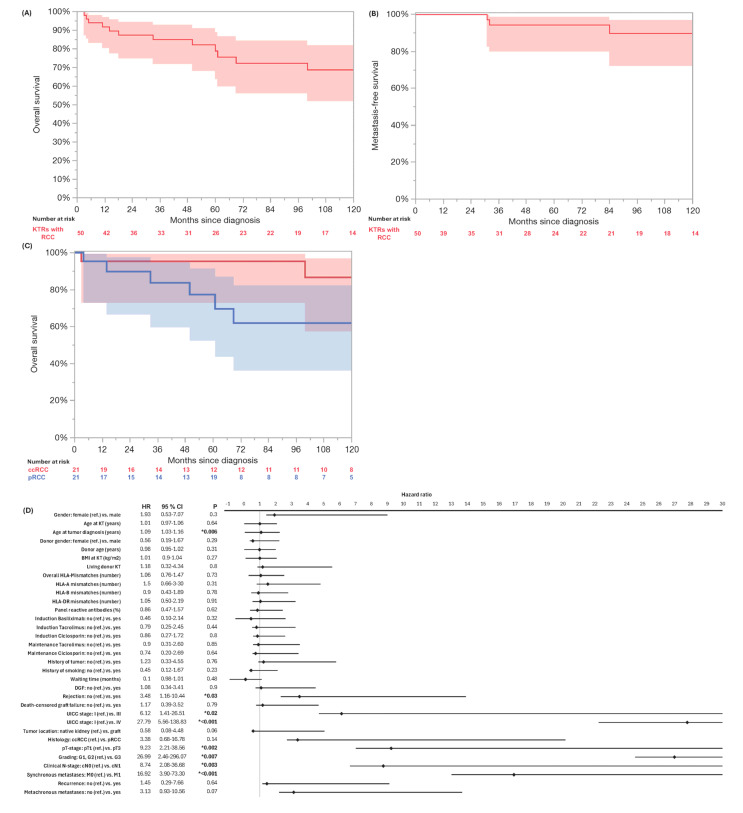
Kaplan–Meier analysis of overall survival (**A**) and metastasis-free survival (**B**) of whole cohort of kidney transplant recipients (KTRs) with de novo renal cell carcinoma. Kaplan–Meier analysis and log-rank testing indicated significant inferior OS in UICC stages ≥ III (blue) compared to UICC stage I (red, *p* < 0.001) (**C**). Univariate Cox regression identified acute rejection (HR: 3.48, 95% CI: 1.16–10.44, *p* = 0.03), older age at RCC (HR: 1.09, 95% CI: 1.03–1.16, *p* = 0.006), advanced UICC stage III (HR: 6.12, CI: 1.41–26.51, *p* = 0.02) and IV (HR: 27.79, CI: 5.56–138.83, *p* < 0.001), higher pT stage (HR: 9.23, 95% CI: 2.21–38.56, *p* = 0.002), cN1 (HR: 8.74, 95% CI: 2.08–36.68, *p* = 0.003), M1 (HR: 16.92, 95% CI: 3.90–73.30, *p* < 0.001), and high-grade tumors (G1/G2 vs. G3, HR: 26.99, 95% CI 2.46–296.07, *p* = 0.007) as significantly associated with poor overall survival (**D**). Significant *p* values are indicated by * *p* < 0.05.

A local recurrence occurred in four patients (8%) after 49.5 (IQR: 31.25–133.75) months. Metachronous metastases developed in four patients (9.3% of M0 cases). The Kaplan–Meier survival analysis is shown in Figure 1. The 3- and 5-year MFS rates were 94%, respectively, and the 3- and 5-year RFS rates were 94% and 90%. Fourteen patients (28%) died during the follow-up. RCC accounted for 3 (6%) deaths, other malignancies for 2 (4.1%) cases, and pneumonia, cardiac arrest, and sepsis for 1 (2%) case, respectively. The median OS for the whole cohort was 199 months (95% CI: 103–295). The 3-year OS was 85%, and the 5-year OS was 72% (Table 3). The OS for the M1-patients was 14 months (95% CI: 0–28), while the OS for those with metachronous metastases was 61 months (CI: 0–126). The Kaplan–Meier analysis indicated a significant inferior OS in higher UICC stages (*p* < 0.001), but no significant differences in the OS between ccRCC and pRCC in the log-rank testing (*p* = 0.11). The univariate Cox regression identified acute rejection (HR: 3.48, 95% CI: 1.16–10.44, *p* = 0.03), older age at RCC (HR: 1.09, 95% CI: 1.03–1.16, *p* = 0.006), advanced UICC stage (HR: 27.79, CI: 5.56–138.83, *p* < 0.001), higher pT stage (HR: 9.23, 95% CI: 2.21–38.56, *p* = 0.002), cN1 (HR: 8.74, 95% CI: 2.08–36.68, *p* = 0.003), M1 (HR: 16.92, 95% CI: 3.90–73.30, *p* < 0.001), and high-grade tumors (G1/G2 vs. G3, HR: 26.99, 95% CI 2.46–296.07, *p* = 0.007) as significantly associated with a poor OS (Figure 1).

### 3.5. Comparison to a Non-Transplant RCC Cohort

**Figure 2:**
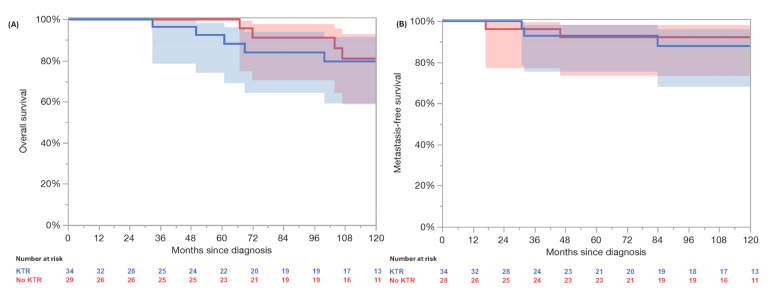
Propensity matched comparison revealed no significant differences in overall survival (**A**) and metastasis-free survival (**B**) between localized renal cell carcinoma patients without kidney graft (red, no KTR) and kidney transplant recipients with localized renal cell carcinoma (blue, KTR) in log-rank testing (*p* = 0.72 and 0.61).

In the PSM cohort without synchronous metastases (34 KTRs, 34 non-KT), no significant differences were observed in age, histological subtype, tumor stage (pT), or grading (Table 4). Three (10%) patients in the non-KTR group and four (11.8%) patients in the KTR group developed metachronous metastases. The log-rank testing revealed no significant differences in the 3-year (100% vs. 96%) and 5-year (96% vs. 84%) OS (*p* = 0.72) and the 3-year (92% vs. 93%) and 5-year (92% vs. 93%) MFS (*p* = 0.61) (Figure 2). The five-year RMST OS analysis revealed 58.6 months (95% CI: 56.7–60.6) in the KTRs and 60.0 months (95% CI: 60.0–60.0) in the non-KTRs, resulting in a non-significant difference of −1.4 months (95% CI: −3.4 to 0.6; *p* = 0.18). At 10 years, the RMST was 108.6 months (95% CI: 99.0–118.2) in the KTRs and 114.0 months (95% CI: 108.0–120.1) in the non-KTR cohort, with a difference of –5.5 months (95% CI: −16.8 to 5.9; *p* = 0.35). Furthermore, the RMST MFS at 5 years was 58.0 months (95% CI: 55.2–60.7) in the KTRs and 57.8 months (95% CI: 54.5–61.1) in the non-KTR cohort, with a non-significant difference of +0.2 months (95% CI: −4.1 to 4.5; *p* = 0.94). At 10 years, the RMST was 111.9 months (95% CI: 103.0–120.8) in the KTRs compared to 113.1 months (95% CI: 103.8–122.4) in the controls, yielding a non-significant difference of −1.2 months (95% CI: −14.1 to 11.7; *p* = 0.86).

## 4. Discussion

The incidence of 0.63 per 1000 person-years and the SIR of 4.40 for RCC in the KTRs in our study aligns with the previous reported incidences of 0.58–0.93%, confirming the elevated risk compared to the general population [4,5,10,11]. Our results showed a comparable SIR compared to the large Transplant Cancer Match Study in the United States, with an SIR of 5.68 (95% CI, 5.27–6.13). Moreover, our study demonstrated a higher risk of pRCC than ccRCC for the KTRs (SIR 12.3 vs. 2.3), which is again consistent with the study, reporting SIRs of 13.3 vs. 3.98 [5]. Our findings are also consistent with the results of the meta-analysis conducted by Crocerossa et al., reporting pRCC and ccRCC rates of 40–41%, respectively [12]. These findings support the hypothesis that the biological mechanisms contributing to the development of renal cell carcinoma in CKD patients may differ from the general population, with the role of long-term dialysis, acquired renal cysts, and, in the case of KT, long-term immunosuppression not yet sufficiently understood [5,13]. Consistent with other studies, the majority of RCCs occurred in the native kidneys rather than the graft, emphasizing the need for the long-term surveillance of the graft and the native kidneys [5,11,14]. The median time from the KT to the RCC diagnosis of 47 months, with a wide range of 0–172 months, also implies that follow-up with attention to RCC after KT should be performed over a long period of time—for example, with annual ultrasound examinations or CT [7,15]. Although the majority of the RCCs in our cohort were detected at low tumor stages, the large subset of advanced tumor growth (pT3) and synchronous metastatic disease (14%, respectively) may indicate a risk for rapid progression if not detected early [16]. In this context, immunosuppression with calcineurin inhibitors (CNIs) such as tacrolimus—which is controversial in the literature—could be a tumor-inducing or growth-promoting factor by inhibiting DNA repair and apoptosis pathways [13]. Since a large proportion of patients in our cohort also received tacrolimus (64%) as maintenance CNI immunosuppression, this could explain the distribution of either low pT1 or advanced stages that we recorded.

The primary treatment modality for localized RCC in our cohort remained surgery, with a native nephrectomy performed in most cases. This is concordant with existing literature [1,4,14]. For RCC in the graft, a partial transplant nephrectomy was attempted in only one case. A systematic review and meta-analysis by Crocerossa et al. highlights that nephron-sparing approaches may be safe and effective options for RCC in transplant kidneys, particularly for tumors classified as pT1a or pT1b. However, their long-term oncological safety remains insufficiently studied. Notably, the OS was significantly shorter after a graft nephrectomy compared to a partial nephrectomy [12]. Recently, we reported two patients who had undergone nephron-sparing surgery for localized pT1a tumors with subsequent stable renal function and without RCC recurrence [8]. This supports the preference for graft-preserving surgical therapy whenever possible [7,17]. However, apart from case reports, clinical data investigating the oncologic outcomes remain scarce. Minimally invasive treatments, such as radiofrequency ablation, were underutilized in our cohort despite being viable options for small renal masses in non-KTRs [18]. With regard to KTRs, Crocerossa et al. were even able to show that there was no inferiority in the OS compared to the partial nephrectomy [12].

The median OS of 199 months in our cohort indicates that RCC in KTRs does not necessarily confer a worse prognosis when managed appropriately [19]. Moreover, the 5-year OS rates of 72% in our cohort align with the previously reported data of KTR cohorts ranging from 55% to 93% [12,13,20,21,22]. The results of the univariate Cox regression analysis indicate that tumor-related factors, particularly advanced stage, metastases, and high-grade histology, are primary OS determinants. KT-specific factors showed no statistical significance in the present study, except for a history of acute rejection. The association between acute graft rejection and poor overall survival could indicate a possible systemic inflammatory component or indirect immunological dysregulation as a prognostic risk factor in RCC patients. Due to the small sample size and limited number of events, a potential prognostic role of KT- and immunosuppression-related factors cannot be excluded and requires further investigation in larger studies. Nevertheless, our matched comparison of localized RCC in KTR and non-KT patients suggests that the OS in KTRs is not significantly inferior. This is concordant with Miao et al., who reported similar OS rates between KTRs with RCC of the Israel Penn International Transplant Tumor Registry and non-KT RCC patients from the Surveillance, Epidemiology, and End Results database [6]. Furthermore, no significant difference in the RFS and MFS between both matched groups was observed in our cohort, indicating that the presence of a graft and corresponding immunosuppression is not a risk factor for the recurrence or development of metastases. Overall, the development of metachronous metastases in 8% of our whole RCC-KTR cohort was low, as the development of nodal or distant metastases during follow-up is described as up to 30% of patients treated with a partial or radical nephrectomy for localized RCC [23]. This highlights the importance and efficacy of surgical therapy in KTRs with localized RCC and may reflect the impact of regular post-transplant imaging, leading to earlier intervention [12,17]. Therefore, based on our institutional practice and current recommendations, we propose a structured long-term surveillance strategy for early tumor detection in KTRs, including annual abdominal ultrasound examinations of the native kidney and the graft [24]. In the case of unclear findings, contrast-enhanced ultrasound should be considered as a further non-invasive diagnostic procedure, if available [25]. Given the late onset of RCC in most transplant recipients, surveillance should be continued beyond the early post-transplant years. However, the effectiveness and cost-efficiency of such protocols warrant further evaluation in prospective studies.

In metastatic RCC, systemic therapy remains challenging due to immune-related risks to the allograft and no existing consensus for systemic treatment [7]. TKIs were the preferred systemic therapy in over 70% of our cohort, reflecting the concern of an increased risk of graft rejection under immunotherapy, as reported by Cui et al. [26]. Notably, patients with metastatic RCC had a significantly inferior survival rate compared to non-metastatic patients. The median OS of 14 months in the synchronous metastatic patients in our cohort appears to be inferior compared with the non-KT patients receiving TKIs, which are reported to be 26.4–28.4 months, underscoring the challenge of treating metastatic disease in KTRs [27]. Therefore, our results are concordant with Miao et al., identifying KT as a risk factor for inferior OS in metastatic RCC [6]. As immune complex-induced nephropathy has been described in addition to the risk of rejection, immunotherapy is not recommended for KTRs [7,26]. One treatment strategy for metastatic RCC in KTRs may include a cytoreductive nephrectomy prior to TKI-based systemic therapy, as performed in one case in our cohort [28]. However, the indication for a cytoreductive nephrectomy must be assessed on a case-by-case basis, considering factors such as the performance status, metastatic burden, and graft function [29]. Future research should focus on developing a safe integration of effective systemic therapies while preserving graft function. One consideration is that mTOR inhibitors may offer dual benefits in KTRs by both suppressing immune responses and exerting anti-tumor effects, though this has not been widely implemented in standard care as antineoplastic therapy [30]. However, mTOR inhibitors, such as Everolimus or Sirolimus, could be incorporated into the immunosuppressive regimen to exert their antineoplastic properties as an additive effect [19,30]. As noted above, in the general population, TKIs in combination with checkpoint inhibitors have shown superior outcomes. However, due to the high risk of graft rejection associated with immunotherapy, TKI monotherapy remains the cornerstone of treatment for metastatic RCC in KTRs [31]. VEGF inhibitors seem particularly suitable, as the VEGF signaling pathway is upregulated in ccRCC due to Von Hippel–Lindau tumor suppressor gene inactivation [28]. The last option may be graft nephrectomy to stop immunosuppressive therapy and start treatment with checkpoint inhibitors [32].

The present study has several limitations. First, the single-center retrospective analysis may limit the generalizability. Second, the sample size is relatively small, which may restrict the statistical power. In particular, the lack of statistically significant differences in OS between the KT-related covariates and histological subtypes, or between the KTRs and non-KTRs, should be interpreted with caution, as these results may be influenced by limited statistical power and do not necessarily reflect a true absence of association. To address these questions more robustly and validate potential prognostic factors, well-powered multicenter studies with harmonized data collection will be essential in the future. Moreover, the assessment of the RCC incidence should be interpreted with caution. Additionally, despite the PSM to balance the differences between the KTRs and non-KTRs, residual confounding factors cannot be ruled out. Particularly, lead time bias must be considered as a potential limitation with an effect on the OS and MFS. In addition, treatment strategies for mRCC have evolved significantly between 2005 and 2024, including the introduction of checkpoint inhibitors and combination therapies as first-line treatments. Therefore, definitive conclusions regarding the OS should be interpreted with care.

## 5. Conclusions

The present study confirms that KTRs have a significantly increased risk of RCC, predominantly in the native kidney and with localized stages, but with a comparable OS and MFS compared to non-KT RCC patients. Advanced stage, cN1, M1, high grade, and rejection after KT are the strongest predictors of poor survival. The management of metastatic RCC in KTRs remains challenging, with limited therapeutic options. These findings underline the importance of long-term surveillance and individualized treatment decisions in KTRs with RCC.

## Figures and Tables

**Table 1:** Patient characteristics and graft outcomes.

Characteristic	RCC (*n* = 50)
Age at KT (years)	56.5 (45.25–63.25)
BMI at KT (kg/m^2^)	23.9 (22.61–27.29)
Gender, *n* (%)	
Male	35 (70)
Female	15 (30)
Donor age (years)	54 (45–62)
Donor gender, *n* (%)	
Male	24 (48)
Female	24 (48)
Unknown	2 (4)
Living donor, *n* (%)	12 (24)
Overall HLA mismatches, *n* (%)	
0	7 (14)
1	3 (6)
2	14 (28)
3	8 (16)
4	9 (18)
≥5	6 (12)
Unknown	3 (6)
HLA-A mismatches, *n* (%)	
0	19 (38)
1	20 (40)
2	8 (16)
HLA-B mismatches, *n* (%)	
0	15 (30)
1	20 (40)
2	12 (24)
HLA-DR mismatches, *n* (%)	
0	15 (30)
1	19 (38)
2	13 (26)
Panel reactive antibodies, *n* (%)	
0	36 (72)
1–20%	1 (2)
21–50%	1 (2)
Unknown	12 (24)
Location of renal transplant, *n* (%)	
Left iliac fossa	24 (48)
Right iliac fossa	26 (52)
Underlying disease for CKD, *n* (%)	
IgA nephropathy	8 (16)
Idiopathic membranous glomerulonephritis	6 (12)
FSGS	6 (12)
Diabetic nephropathy	4 (8)
ADPKD	1 (2)
Hypertensive kidney disease	4 (8)
Nephrosclerosis	5 (10)
Renal atrophy	5 (10)
Tubulointerstitial nephritis	4 (8)
Other	7 (14)
Induction immunosuppression	
Basiliximab	42 (84)
Janus kinase inhibitor	1 (2)
Thymoglobuline	1 (2)
Infliximab	1 (2)
Unknown	4 (8)
Maintenance immunosuppression, *n* (%)	
Tacrolimus	32 (64)
MMF	47 (94)
Ciclosporin	10 (20)
Corticosteroids	26 (52)
Waiting time for KT (months)	36.5 (10.75–77.25)
Time of dialysis (months)	62.5 (21.5–88.75)
Remaining diuresis prior KT (ml)	300 (0–1500)
Type of dialysis, *n* (%)	
Hemodialysis	46 (92)
Peritoneal dialysis	3 (6)
CIT (min)	667 (187.25–989.75)
Operative time (min)	216 (181.25–251.25)
Major complication, *n* (%)*;* (CDC ≥ 3, 30 d)	5 (10)
Delayed graft function, *n* (%)	20 (40)
Rejection, *n* (%)	15 (30)
Creatinine level (mg/dL)	
Preoperative	7.1 (5.86–9.24)
6 months post KT	1.5 (1.22–2.03)
1 year post KT	1.5 (1.10–2.00)
3 years post KT	1.6 (1.14–2.00)
5 years post KT	1.6 (1.20–2.04)
Graft failure	22 (44)
Death censored graft failure, *n* (%)	16 (32)
Cause of graft failure, *n* (%)	
Chronic rejection	5 (10.9)
Tumor	2 (4.3)
Nephrosclerosis	1 (2.2)
Primary non function	1 (2.2)
Acute kidney injury	1 (2.2)
Death with functioning graft	6 (13)
CNI toxicity	1 (2.2)
Unknown	2 (4.3)
Transplant nephrectomy, *n* (%)	3 (9.1)
Follow-up after KT (months)	139 (88.25–175)

Abbreviations: RCC: renal cell carcinoma; BMI: body mass index; KT: kidney transplantation; CIT: cold ischemia time; MMF: mycophenolate mofetil; CKD: chronic kidney disease; ADPKD: autosomal dominant polycystic kidney disease; FSGS: focal segmental glomerulosclerosis; HLA: human leucocyte antigen. Estimates were given as median (interquartile range) or frequency (percentage).

**Table 2:** Tumor-specific patient characteristics and oncologic features.

Characteristic	RCC (*n* = 50)
Age at tumor diagnosis (years)	58.5 (51–68.25)
Time from KT to tumor (months)	47 (13.25–83.50)
Prior malignancy, *n* (%)	11 (22)
History of smoking, *n* (%)	18 (36)
Hematuria, *n* (%)	3 (6)
Tumor location, *n* (%)	
Native kidney	46 (92)
Kidney transplant	4 (8)
Unilateral	48 (96)
Bilateral	2 (4)
Tumor number, *n* (%)	
1	45 (90)
2	5 (10)
Histology, *n* (%)	
ccRCC	21 (42)
pRCC	21 (42)
ccRCC + pRCC	2 (4)
Others	4 (8)
Unknown	2 (4)
UICC stage, *n* (%)	
I	37 (74)
III	6 (12)
IV	7 (14)
pT-Stadium, *n* (%)	
1a	28 (56)
1b	9 (18)
3a	5 (10)
3b	1 (2)
3c	1 (2)
Unknown	6 (12)
Grading, *n* (%)	
G1	10 (20)
G2	23 (46)
G3	7 (14)
G4	1 (2)
Unknown/unclear	9 (18)
Clinical positive lymph nodes, *n* (%)	8 (16)
Synchronous metastases (M1), *n* (%)	7 (14)
Pulmonary	6 (85.7)
Lymphonodal	5 (71.4)
Bone	4 (57.1)
Hepatic	1 (14.3)
IMDC low risk	3 (42.9)
IMDC high risk	4 (57.1)
Follow-up after tumor diagnosis (months)	60.5 (21–131.75)

Abbreviations: RCC: renal cell carcinoma; ccRCC: clear cell renal cell carcinoma; pRCC: papillary renal cell carcinoma; KT: kidney transplantation; IMDC: International Metastatic RCC Database Consortium score. Estimates were given as median (interquartile range) or frequency (percentage).

**Table 3:** Therapy regimes and oncologic outcomes.

Characteristic	RCC (*n* = 50)
Therapy—non-metastatic RCC (M0), *n* (%)	
None	1 (2.3%)
Native nephrectomy	36 (83.7)
Native nephrectomy + TKI	1 (2.3)
Transplant nephrectomy	3 (7)
Partial transplant nephrectomy	1 (2.3)
Local ablation	1 (2.3)
Therapy—synchronous metastatic RCC (M1), *n* (%)	
Native nephrectomy	2 (28.6)
TKI	4 (57.1)
Native nephrectomy + TKI	1 (14.3)
R-Status, *n* (%)	
R0	38 (76)
R1	2 (4)
Recurrence, *n* (%)	4 (8)
Contralateral native kidney	3 (75)
Contralateral native kidney and graft	1 (25)
Time to recurrence (months)	49.5 (31.25–133.75)
Recurrence-free survival	Median not reached
3-year RFS	94%
5-year RFS	90%
Metachronous metastasis, *n* (%)	4 (8%)
Pulmonary	2 (50%)
Lymphonodal	3 (75%)
Bone	1 (25%)
Retroperitoneal	1 (25%)
Time to metastases (months)	58 (31.25–143.25)
Metastasis-free survival	Median not reached
3-year MFS	94%
5-year MFS	94%
Death, *n* (%)	14 (28%)
Cause of death, *n* (%)	
RCC	3 (6%)
Other malignancy	2 (4.1%)
Pneumonia	1 (2%)
Sepsis	1 (2%)
Cardiac arrest	1 (2%)
Unknown	6 (12.2%)
Overall survival—whole cohort (months)	199 (CI 103–295)
3-year OS	85%
5-year OS	72%
OS with synchronous metastases (months)	14 (CI 0–28)
OS with metachronous metastases (months)	61 (CI 0–126)

Abbreviations: RCC: renal cell carcinoma; TKI: tyrosine kinase inhibitor; OS: overall survival; RFS: recurrence-free survival; MFS: metastasis-free survival. Estimates were given as median (interquartile range or confidence interval) or frequency (percentage). Survival analysis was performed with Kaplan–Meier method.

**Table 4:** Characteristics and outcomes of propensity score-matched cohort of localized RCC patients with and without prior kidney transplantation.

Characteristic	Non-KTR with RCC (*n* = 34)	KTR with RCC (*n* = 34)	SMD	*p* *
Age (years)	58 (50.75–65)	56.5 (47–66.5)	0.05	0.91
Histology, *n* (%)			<0.001	1.0
ccRCC	18 (52.9)	18 (52.9)		
pRCC	16 (47.1)	16 (47.1)		
pT-Status, *n* (%)			0.26	0.76
1a	24 (70.6)	22 (64.7)		
1b	7 (20.6)	8 (23.5)		
3a	3 (8.8)	3 (8.8)		
3b	0	1 (2.9)		
Grading, *n* (%)			0.11	0.34
G1	7 (20.6)	8 (23.5)		
G2	26 (76.5)	22 (64.7)		
G3	1 (2.9)	4 (11.8)		
Metachronous metastases, *n* (%)	3 (10)	4 (11.8)		0.82
Metastasis-free survival	Median not reached	Median not reached		0.61
3-year	92%	93%		
5-year	92%	93%		
Restricted mean metastasis-free survival time (months)				
5-year	57.8 (CI 54.5–61.1)	58 (CI 55.2–60.7)		0.94
10-year	113.1 (CI 103.8–122.4)	111.9 (CI 103–120.8)		0.86
Death, *n* (%)	5 (16.1)	6 (17.6)		0.87
Overall survival (months)	Median not reached	199 (CI 60–338)		0.72
3-year	100%	96%		
5-year	96%	84%		
Restricted mean overall survival time (months)				
5-year	60 (CI 60–60)	58.6 (CI 56.7–60.6)		0.18
10-year	114 (CI 108–120.1)	108.6 (CI 99–118.2)		0.35

Abbreviations: RCC: renal cell carcinoma; ccRCC: clear cell renal cell carcinoma; pRCC: papillary renal cell carcinoma; KTR: kidney transplant recipient; SMD: standard mean difference. * *p* values were calculated using Mann–Whitney U test for continuous variables and Chi-square test for categorical variables. Survival analysis was performed with Kaplan–Meier method and log-rank test. Estimates were given as median (interquartile range or confidence interval) or frequency (percentage).

## Data Availability

The data that support the findings of this study are available on request from the corresponding author.

## Abbreviations

The following abbreviations are used in this manuscript:

BMI	Body Mass Index
CDC	Clavien–Dindo Classification
CI	Confidence Interval
CNI	Calcineurin Inhibitors
CKD	Chronic Kidney Disease
CIT	Cold Ischemia Time
ccRCC	Clear Cell Renal Cell Carcinoma
cN1	Clinical Nodal Involvement
DGF	Delayed Graft Function
ESRD	End-Stage Renal Disease
GF	Graft Failure
GS	Graft Survival
HR	Hazard Ratio
KT	Kidney Transplantation
KTRs	Kidney Transplant Recipients
M1	Metastatic Disease
MMF	Mycophenolate Mofetil
MFS	Metastasis-Free Survival
OS	Overall Survival
PSM	Propensity Score Matching
pRCC	Papillary Renal Cell Carcinoma
pT	Pathologic Tumor Stage
RCC	Renal Cell Carcinoma
RMST	Restricted Mean Survival Time
SMD	Standard Mean Difference
TKI	Tyrosine Kinase Inhibitor
UICC	Union Internationale Contre le Cancer
